# Feeling the Fit: Emotional Association of Vocational Interest and Indecision in Youth

**DOI:** 10.1177/10690727251396109

**Published:** 2025-11-07

**Authors:** Shékina Rochat, Alexia Gaillard, Elise Dan-Glauser

**Affiliations:** 1 Institute of Psychology, University of Lausanne, Lausanne, Switzerland; 2 Faculty of Psychology, UniDistance Switzerland, Brig-Glis, Switzerland; 3 FORS Swiss Centre of Expertise in the Social Sciences, Lausanne, Switzerland

**Keywords:** dispositional affect, situational emotions, mixed emotions, vocational interests, vocational indecision

## Abstract

The role of emotions is understudied in relation to career choices. Building on prior research suggesting that emotions can shape decision-making and interest, this study uses an experimental design to investigate the emotions evoked by various career options and their relationship with vocational interests and indecision. Fifty-seven youths participated in the experiment, which involved identifying the interests and emotions elicited by professional activities represented in photographs, after completing a questionnaire on indecision and dispositional affects. Results show that over half of the occupational photos triggered at least one positive or negative emotion. Additionally, one-fourth of the photos evoked mixed emotions (both positive and negative). Specific positive and negative emotions were distinctly related to (dis)interest in RIASEC domains. Dispositional affects, but not situational ones, were significantly associated with indecision—with the notable exception of mixed emotions in the Social domain, which were linked to internal conflicts. Implications for practice and research are discussed.

The world of work is increasingly marked by uncertainty, making career decision for young people more challenging. Sometimes at a very young age, individuals are faced with a growing number of career choices (e.g., Borgen & Edwards, 2019; Savickas et al., 2009). Making such choices is a complex process that can be hindered by vocational indecision, defined as “problems individuals may have in making their career decision” (Gati et al., 1996, p. 510). To support individuals in navigating these decisions, numerous models have been developed to describe the key steps (e.g., Gati & Asher, 2001; Sampson et al., 1999) and essential ingredients (e.g., Dawis & Lofquist, 1984; Holland, 1959) of career decision-making. In addition, several taxonomies of decision-making difficulties have been proposed (see Xu & Bhang, 2019, for a review).

These models assume that career choice is a rational process—one that can be optimized by identifying the relevant steps to follow and the specific vocational problems to solve (Xu, 2023). This perspective aligns with the concept of *homo economicus*, which posits that individuals make rational choices, grounded in a clear understanding of their preferences (assumed to be stable and well-structured), as well as of the available alternatives and their consequences (assumed to be finite). According to this view, individuals are then capable of calculating which options will best satisfy their preferences (Simon, 1955). However, several findings have challenged this rational perspective. Simon (1955) showed that individuals are not as rational as once thought. Moreover, Kahneman and Tversky (1979) identified numerous cognitive biases that disrupt the decision-making process. Finally, the work of Damasio (1994) highlighted the essential role of emotions in decision-making.

As a result, researchers have increasingly focused on the role of emotions in various aspects of vocational development and behavior, including vocational identity (Garrison et al., 2017), career adaptability and professional commitment (Hartung et al., 2022), indecisiveness (Farnia et al., 2018), career choice self-efficacy (Betz et al., 2005; Larson & Majors, 1998; Park et al., 2019, 2021), career well-being (Kidd, 2008), job search behavior (Kim & Lee, 2022), career counseling interactions (Olry-Louis, 2018), choice anxiety (Park et al., 2019), career-related actions (Young et al., 1997), and the anticipation of career transition (Parmentier et al., 2021, 2022, 2023; Vignoli et al., 2019). Focusing more specifically on the role of emotions in career decision-making, several authors explored the link between emotional state (often considered as propensities to feel certain states) and vocational indecision (Betz et al., 2005; Hartung et al., 2022; Meldahl & Muchinsky, 1997; Multon et al., 1995; Park et al., 2021). Furthermore, Puffer (2015) showed that the wording used in career assessment instruments can itself act as a stimulus capable of triggering emotional responses.

While these results underscore the relationship between emotions and the career decision-making process, existing research protocols present several limitations. First, the studies mentioned above (Betz et al., 2005; Hartung et al., 2022; Meldahl & Muchinsky, 1997; Multon et al., 1995; Park et al., 2021) primarily relied on self-reported instruments to assess emotions such as the Positive and Negative Affect Schedule (PANAS; Watson et al., 1988). Moreover, the positive and negative affects measured in these studies reflected general emotional experience, rather than emotions specifically tied to the career choice process. Given that emotions are elicited by specific circumstances depending on their relevance to personal goals (M. E. Ford & Smith, 2020), and that emotions with the same valence (i.e., positive or negative) can arouse different responses—such as promoting or inhibiting action (Pekrun et al., 2023)—examining general affective states in relation to career decision-making appears too vague.

Second, with the notable exception of Puffer (2015)—who examined the type of emotions elicited by the name of preferred career options—none of these studies investigated the role of emotions in shaping expressed career interests. This gap is unfortunate because it obscures our understanding of how specific emotions relate to the development and expression of vocational interests and career choices (e.g., Lent et al., 1994; Vondracek et al., 2014). Third, these studies mostly focused on the separate correlations between positive and negative emotions and vocational indecision, without considering the role of mixed emotions. Yet, individuals often experience such emotional ambivalence when anticipating future career transitions (Parmentier et al., 2021, 2022, 2023; Vignoli et al., 2019), and it is plausible that these conflicting emotional states influence the affective valence attributed to different activities—thereby shaping the career decision-making process.

The present paper aims to address these limitations by investigating the role of emotions in career choice through an experimental design. Specifically, we examine both interests and emotional responses elicited by photographs depicting individuals engaged in various professional activities and assess the relationships between these emotional and interest-based evaluations, as well as their association with vocational indecision. This work extends current knowledge on the emotional aspects of career decision-making in three specific ways. First, we introduce an innovative research protocol that is better suited to capturing the emotional dimensions of career decision-making than general self-report-based methods. Second, we build on Puffer’s (2015) findings by showing the variety of emotions triggered by different career options and their relationship to expressed interest. Finally, we promote a more nuanced understanding of the role of emotions in career choice, particularly by exploring the influence of mixed emotions and the role of emotions in expressed interest.

## Theoretical Background and Hypothesis Development

### Emotions and Vocational Imagination

Recently, Vondracek et al. (2014) adapted D. H. Ford’s (1987) Living System Framework (LSF), and his son M. E. Ford’s (1992) Motivational System Theory into the Living Systems Theory of Vocational Behavior and Development (LSTVBD). In the LSF, a *behavior episode* (BE) refers to a momentary sequence of goal-directed and context-specific behavior carried out by a person over time. The processes involved in such person-in-context functioning include goals, beliefs, emotions, skills, biological structures and functioning, and contextual factors. Through repeated experiences of similar behavior episodes, individuals develop integrated internal representations known as behavioral episode schemata (BES). Those contribute to the formation of enduring traits such as personality and competence. In this framework, emotions—which can be defined as a “complex reaction pattern, involving experiential, behavioral, and physiological elements, by which an individual attempts to deal with personally significant matter or event” (American Psychological Association, 2015, p. 362)—play a central role: they help evaluate the desirability and attainability of goals and provide the energy to pursue them.

Emotions are thus present in every behavioral episode, whether the episode is instrumental (i.e., direct action), observational (i.e., watching others) or imaginative (i.e., mental simulation.) In particular, imaginative behavior episodes are seen as essential “cognitive activities in preparation for—and selection of—future possibilities” (Vondracek et al., 2014, p. 44). These episodes allow individuals to mentally explore potential futures and guide their actions. Building on this theory, we propose that imagining oneself engaged in an occupational activity depicted in a photo will elicit emotional responses. This is supported by findings from Puffer (2015), who showed career-related words used in assessment tools can evoke a range of emotions. More precisely, drawing on prior research on the emotional anticipation of future career transitions (Parmentier et al., 2021, 2022, 2023), we hypothesize that these emotions may be positive, negative, or mixed (i.e., the simultaneous occurrence of both positive and negative emotions).

Hypothesis 1a Imagining oneself performing the occupational activities depicted in photos will elicit emotional responses.

Hypothesis 1b The emotions elicited by imagining oneself performing these occupational activities may be positive, negative or mixed.

### Emotions and Vocational Interests

The LSTVBD posits that individuals tend to seek the repetition of behavior episodes that have previously elicited positive emotions, and are therefore more likely to pursue career paths that involve behaviors associated with such emotions (Vondracek et al., 2014). Accordingly, in this framework emotions play a central role in the formation and development of vocational interests. In fact, vocational interests have themselves been conceptualized as emotional in nature (Barak, 2001): a perspective echoed in the distinction between *situational* and *dispositional* interests:

“Situational interest is defined as the context-specific state of emotional experience, curiosity, and momentary motivation [while], dispositional interest is trait-like, reflecting a person’s preferences for behaviors, situations, contexts in which activities occur, and/or the outcomes associated with the preferred activities” (Su et al., 2009, p. 860).

Following this view, Barak (2001) suggests that assessment of vocational interest should include an examination of the emotional antecedents and correlates of interest across a range of stimulus contents. Based on these considerations, we propose that experience of positive emotions elicited by imagining oneself performing specific occupational activities will be associated with vocational interest, both for the specific activity depicted in the photo, and for the broader RIASEC domain of vocational interests (Realistic, Investigative, Artistic, Social, Enterprising, and Conventional; Holland, 1959) that the activity represents.

Given that different emotions are characterized by distinct subjective experiences, physiological responses, and motivational functions (M. E. Ford & Smith, 2020), we propose that different types of positive emotions (specifically joy, pride and hope) will show differentiated associations with expressed vocational interest in particular career activities. In other words, each emotion may influence vocational interest in distinct ways. Conversely, we hypothesize that the experience of negative emotions (specifically fear, anger, or shame) in response to imagining oneself performing certain occupational activities will be associated with vocational disinterest, both for the specific activity depicted and for the broader RIASEC domain it represents. As with positive emotions, we expect that different negative emotions will relate differently to expressed disinterest for specific career activities. Finally, we hypothesize that the experience of mixed emotions when imagining oneself engaged in a professional activity will neutralize the association with vocational interest, due to the conflicting processes involved, which may weaken or suppress clear preference formation, for both the specific activity and the corresponding RIASEC domain. We thus hypothesize:

Hypothesis 2a Experiencing positive emotions when imagining oneself performing an occupational activity will be positively associated with expressed interest in both the specific activity and its corresponding RIASEC domain.

Hypothesis 2b Different types of positive emotions experienced in response to imagining oneself performing an occupational activity will show differentiated associations with interest in the specific activity and its corresponding RIASEC domain.

Hypothesis 2c Experiencing negative emotions when imagining oneself performing an occupational activity will be negatively associated with expressed interest in both the specific activity and its corresponding RIASEC domain.

Hypothesis 2d Different types of negative emotions experienced in response to imagining oneself performing an occupational activity will show differentiated associations with disinterest in the specific activity and its corresponding RIASEC domain.

Hypothesis 2e Experiencing mixed emotions when imagining oneself performing an occupational activity will weaken the previously observed association between emotional response and expressed interest, both for the specific activity and its corresponding RIASEC domain.

### Emotions and Vocational Indecision

The LSTVBD emphasizes the central role of emotions in career decision-making by providing immediate, compelling, and short-term evaluative information about relevant stimuli, working in tandem with cognitive patterns to help individuals assess the desirability and attainability of pursuing specific career goals (Vondracek et al., 2014). When positive outcomes are anticipated “the physiological and transactional emotion components provide energy to help fuel the processes of acting on one’s intentions. If one anticipates negative outcomes, those components regulate energy as appropriate to the situation (e.g., fight, flight, withdrawal, etc.)” (M. E. Ford & Smith, 2007, pp. 160–161). At the BES level, these considerations align with previous research’s results. Studies have shown that positive dispositional affective states are associated with lower levels of vocational indecision—that is “a state of being undecided about one’s educational, occupational, or career-related career path” (Xu & Bhang, 2019, p. 2)—whereas negative affective states and emotions correlate with higher levels of vocational indecision (Betz et al., 2005; Farnia et al., 2018; Hartung et al., 2022; Meldahl & Muchinsky, 1997; Multon et al., 1995; Park et al., 2021).

After verifying that our findings replicate previous results (namely, that negative dispositional affects are positively associated with vocational indecision, while positive dispositional affects are negatively associated), we will test the hypothesis that dispositional affects also predict the overall emotional experience elicited by photos. According to the LSF, BES provide “guidance (…) about what one should pay attention to and how one should think, feel and act in a specific behavior episode” (M. E. Ford, 1992, p. 127). Building on this, we posit that negative and positive dispositional affects will shape the overall emotional tone of the experience triggered by photos. Furthermore, we hypothesize that the experience of mixed emotions in response to photos will be positively associated with vocational indecision. This assumption is grounded in the concept of decisional ambivalence—defined as “the incidence of contradictory thoughts, feelings, and intentions [when] choosing a [career] path” (Kasperzack et al., 2014, p. 249), or more broadly, as a form of internal conflict (see Gati et al., 1996). Previous research has shown that such ambivalence can hinder the career decision-making process (e.g., Kasperzack et al., 2014). In summary, we hypothesize that:

Hypothesis 3a Negative dispositional affects will be positively associated with vocational indecision, whereas positive dispositional affects will be negatively associated with vocational indecision.

Hypothesis 3b Negative dispositional affects will predict a more negative overall emotional experience in response to the TIP photos, while positive dispositional affects will predict a more positive overall experience.

Hypothesis 3c The experience of mixed emotions in response to the TIP photos will be positively associated with vocational indecision.

## Method

### Participants and Recruitment

The participants were recruited from a youth integration program in Switzerland, designed to support young people who are neither in education, employment, nor training (NEETs), and to offer apprenticeship training programs for IT specialists or office clerks. In this national context, the majority of adolescents follows a vocational track through apprenticeship, which typically begins around the age of 15 (GFS.Bern, 2023). However, due to their young age and the competitive nature of the apprenticeship market, many experience long-term difficulties in securing apprenticeship positions that match their interests or qualifications (GFS.Bern, 2023). The program includes both NEETs and young apprentices who have faced similar difficulties in accessing or completing vocational education. Most of these participants come from disadvantaged or migrant backgrounds (Schmid, 2020), with a notable proportion being girls with lower academic achievement (Perdrix et al., 2012).

In total, 57 young people (56.14% of women) between the ages of 16 and 27 years (*M* = 19.52, *SD* = 2.37) took part in the study, with 68.42% being identified as NEETs. Due to the shared admission criteria for both NEETs and apprentices entering the youth integration program, and given the similar difficulties experimented by both groups, these were combined for analysis.

Program coaches introduced the study to the youth and distributed flyers containing additional information. Approximately 63% of the eligible individuals agreed to participate. The inclusion criteria were as follows: (a) being enrolled in the integration program; (b) being fluent in written and spoken French; (c) having normal or corrected-to-normal vision. The only exclusion criterion was being over 30 years of age. All eligible participants were included in the study. This study was approved by the Research Ethics Commission of the University of Lausanne (number C_SSP_092023_00003).

### Measures

#### Vocational Indecision

Given the relevance of career decision-making difficulties to understanding vocational indecision (Xu & Bhang, 2019), we used the French version of the Career Decision-Making Difficulties Questionnaire (CDDQ; Gati et al., 1996) to assess it. In this scale, 35 items (including two validity items and one general item assessing difficulties in making a career choice) are rated on a Likert scale ranging from 1 (“does not describe me”) to 9 (“describes me well”). Items are grouped into ten types of choice difficulties, which are further grouped into three main categories: (1) lack of preparation (lack of motivation, indecisiveness, and dysfunctional beliefs); (2) lack of information (on the choice process, on oneself, on the options, or on how to obtain additional information); and (3) inconsistent information (presence of unreliable information, internal conflicts, or external conflicts). For the French version, Rossier et al. (2022) reported strong internal consistency, with a Cronbach’s alpha of .92 for the total score. The three dimensions showed alphas ranging from .57 (Lack of Preparation) to .91 (Lack of Information) with a median of .84. For the ten subscales, alphas ranged from .53 (dysfunctional beliefs) to .83 (lack of information about the career choice process) with a median of .72.

#### Dispositional Affects

Dispositional affective tendencies were assessed using a selection of 10 items from the French version (Bouffard & Lapierre, 1997) of the Positive and Negative Affect Schedule (PANAS; Watson et al., 1988). The original PANAS comprises 20 items initially, divided equally into ten positive emotions (PANAS+) and ten negative emotions (PANAS-). The reliability of Bouffard and Lapierre’s (1997) French version was excellent for the PANAS+ subscale (α = .90), and fair for the PANAS- subscale (α = .77). The 10 items used in this study correspond to those retained by Parmentier et al. (2021), specifically five positive anticipatory emotions (excited, strong, enthusiastic, proud, and determined; PANAS+), and five negative anticipatory emotions (jittery, distressed, scared, nervous, and afraid; PANAS-). Parmentier et al. (2021) reported good internal consistency for these shortened subscales (PANAS+: α = .86; PANAS-: α = .87). To facilitate links with the emotions assessed during the experimental phase, three additional items were included: “hopeful”, “ashamed” and “joyful.” Participants were asked to rate the extent to which they generally experience each emotion on a 5-point Likert scale ranging from 1 (“very little or not at all”) to 5 (“extremely”).

#### Interest in Vocational Activities

**Figure 1.**
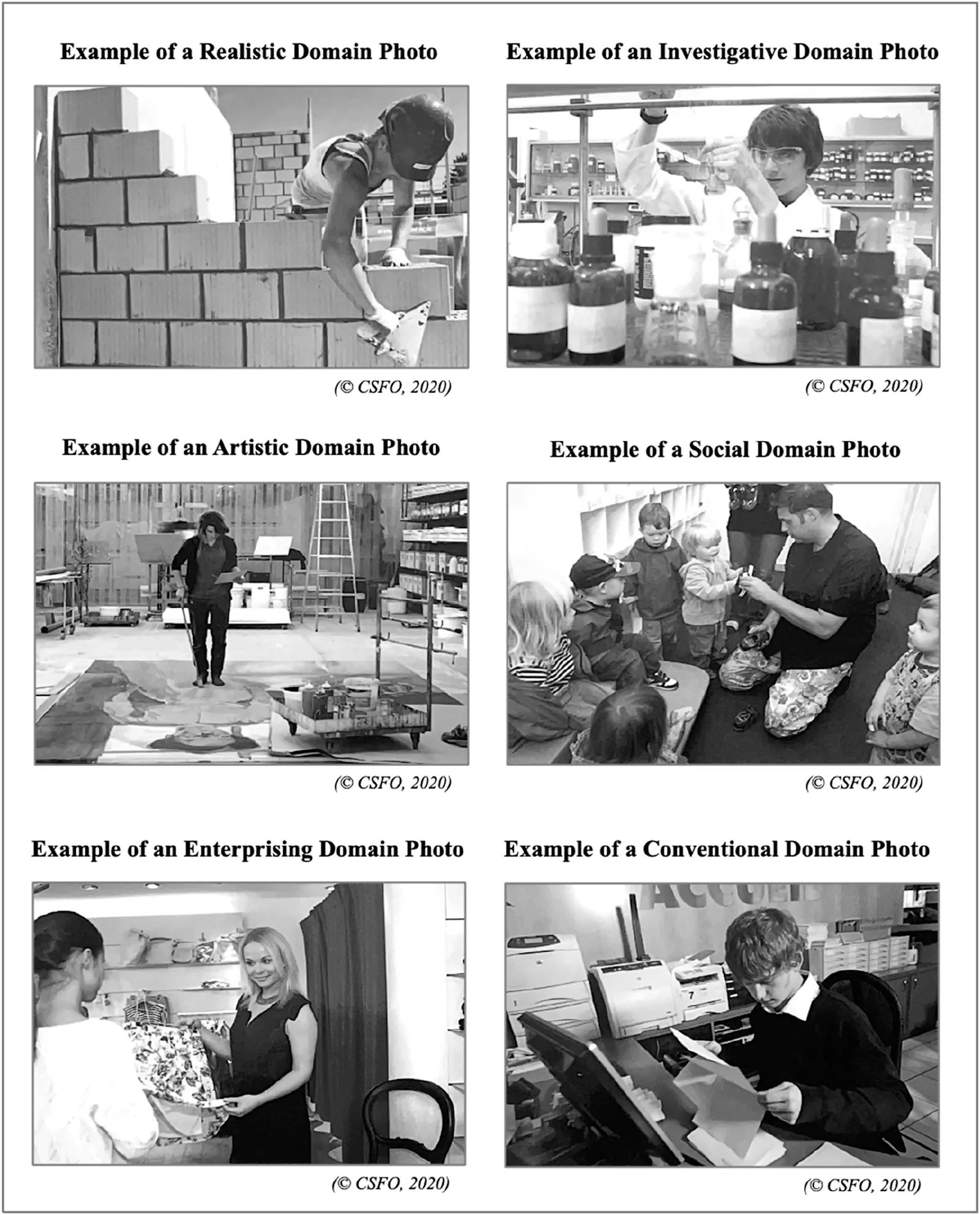
Examples of TIP Photos Selected to Represent Each RIASEC Domain. *Note.* The Original Images Were in Color. Some Visual Details May Not Be Fully Visible in the Black and White Reproductions. These Images are Reproduced With Permission From CSFO (Swiss Centre for Vocational Education and Career Guidance). All Rights Reserved

Vocational interests were assessed using the Photo Interest Test (TIP; Gubler et al., 2019), which consists of 125 standardized photographs depicting individuals engaged in various professional activities, all of which correspond to professions accessible through apprenticeships. Prior to administering the TIP, the researchers confirmed through discussions with program coaches that participants had not been recently exposed to it (i.e., since enrolling in the program). Participants were asked to sort the photos into three categories, based on their level of interest: low, medium or high. Each photo included a code on the back, allowing for the calculation of interest scores across six professional domains. To reduce the length of the protocol, only five photos per domain were selected for this study. The selection was based on their relevance to Holland’s (1959) RIASEC model, as determined by a panel consisting of three qualified career counseling psychologists and one trainee, and by ensuring balanced gender representation across all domains. This assessment was further validated by comparing the represented professions with the RIASEC code provided by the O*net online database. Examples of the photo selection per RIASEC domain are shown in Figure 1. During the session, the researcher presented three cards labeled as “no interest,” “medium interest,” and “strong interest” to the participants. Then the participant was handed the 30 numbered photos, one at a time, and asked to classify each according to their level of interest in the depicted activity. The researcher recorded each response on a data sheet. After all photos were rated, they were collected, reordered numerically (thus removing the interest sorting) and the labels were removed from the table.

#### Situational Affects

To assess situational affective responses to various professional activities, we used the same set of 30 images from the Photo Interest Test (Gubler et al., 2019) as previously described. Each photo was shown individually to the participants, who were asked to indicate which positive emotions they would feel if they were performing the activity. Pre-defined emotions were selected based on two criteria: (1) they had to clearly refer to emotions (as opposed to general feelings, such as “attraction”); and (2) they had to correspond to a possible emotion experienced while performing the depicted action. Response options included: joy, pride, hope, “no positive emotions”, or “other positive emotions.” When “other positive emotions” was selected, participants were prompted to specify the emotion they had in mind. The same process was then repeated for negative emotions. Participants were asked to identify which negative emotions they would feel, choosing among fear, anger, shame, “no negative emotions”, or “other negative emotions”.

#### Demographics

In accordance with ethical guidelines (e.g., European Union, 2016), which require researchers to collect personal data sparingly—focusing only on socio-demographic variables directly relevant to the research hypotheses—only age and gender were collected. No additional socio-demographic information was gathered. While no further data were collected, the nature of the program and its typical target population suggest that participants were likely to come from low socioeconomic backgrounds, possibly with a history of migration and limited educational attainment. This assumption, however, is speculative and should be interpreted with caution.

### Procedure

Data collection was conducted between September and December 2023 at the premises of youth integration program sites. Young people interested in participating registered with the program coaches after attending a short presentation and receiving an information sheet about the study. On scheduled administration days, the coaches directed participants one at a time to the office where the researcher (Author 1 or Author 2) was located. Once seated across from the researcher, participants were given the information sheet again and asked to sign the informed consent form. They were then provided with a tablet and asked to complete an online survey hosted on Qualtrics (Provo, UT). The survey included several quantitative questionnaires, such a measure of vocational indecision, as well as assessments of occupational and activity interests, depression/anxiety, and emotional regulation difficulties (data of the latter three instruments were not included in the present study). Restricted demographic information was also collected at this stage.

After completing the survey, the experimental portion of the study began. It consisted of three rounds of photos sorting tasks using the 30 pre-selected images from the TIP. The photos were presented in the same order for all participants to ensure identical experimental conditions across individuals and to minimize between-subject variability related to uncontrolled random order effects. In the first round, participants indicated their interest in the vocational activities depicted (see “Interest in vocational activities” section). In the second round, the same images were presented again in the same order, and participants were asked to report the positive emotions they would experience if performing the activities (see “Situational affects” section). In the third and final round, participants reported the negative emotions associated with imagining themselves engaged in each activity. The experiment concluded after this final phase. Participants were then given a sheet listing institutional and professional resources, in case the experience triggered difficult emotions. When participants asked for clarification regarding the content of a photo or the meaning of an emotional label, the experimenter provided a standardized, preapproved explanation. These responses were limited to factual descriptions of the term and avoided any evaluative elements that could influence emotional judgments. When participants asked for the specific names of the professions represented by the photos, this information was only provided at the end of the protocol so as not to influence their responses.

### Data Analyzes

First, for each participant, we counted the number of photos associated with a negative emotional response and the number associated with a positive emotional response. These counts were then converted into percentages out of the 30 photos presented. Next, within the set of positive and negative responses, we identified how many corresponded to the predefined emotional categories: anger, shame, and fear for the negative emotions; joy, pride, and hope for the positive. This allowed us to calculate the proportion of each predetermined emotion category among all positive and negative emotional responses, respectively. Additionally, we identified mixed emotional responses (i.e., photos that elicited both a positive and a negative emotion) and documented the frequency of each emotion pair. Given that girls’ specific vocational interests may further reduce their chances of obtaining a suitable apprenticeship (e.g., Perdrix et al., 2012), the frequencies of each emotion pair were further examined separately for males and females.

To test our first hypothesis, we performed one-sample *t*-tests using 0 as the reference point (indicating no emotion reported) for each emotional response type. We then conducted three *t*-tests to compare the frequencies of positive, negative, and mixed responses, as well as to compare the frequencies of the predetermined emotion categories within each valence. The effect size of these differences was calculated using Cohen’s d. A Cohen’s d ≤ .20 was considered small; .50 medium; and ≥0.80 large (Cohen, 1988).

To test our second hypothesis, we calculated the average interest expressed by participants for the TIP photos, based on a 3-point scale: 1 (low), 3 (average), and 5 (high). We computed average interest scores across all 30 photos and separately for each of the RIASEC domains. We then examined the relationship between interest levels and the number of TIP photos that evoked one of the three predefined positive (joy, pride, hope) or negative (fear, anger, and shame) emotions. These analyses were conducted using Pearson’s correlation. To test Hypotheses 2a and 2c, we performed the analysis both globally (i.e., across all 30 photos) and within each RIASEC domain. In addition, to test Hypotheses 2b and 2d, we examined each emotion separately to explore potential differences in how specific emotional responses related to vocational interest. To test Hypothesis 2e, we separated the TIP photos into two categories: those that elicited polarized (i.e., single valence) emotions and those that elicited mixed emotions.

To test Hypothesis 3, we began by testing Hypothesis 3a which aimed to replicate previous findings linking dispositional emotions (as measured with the PANAS) and indecision. We computed Pearson’s correlation between the PANAS subscale scores and the CDDQ scores (including the total score, the three categories, and ten subdimensions). We then performed regression analyses with PANAS- scores as the outcome variable, using only those indecision dimensions that showed significant correlations in the previous step. The same procedure was followed using PANAS+ scores as an outcome to examine their links with vocational indecision. To test Hypothesis 3b, we computed a correlation matrix between dispositional affect scores (PANAS+ and PANAS-) and the TIP-induced emotional responses (positive and negative). Finally, for testing Hypothesis 3c, we explored the correlations between the number of mixed emotions elicited by the TIP photos and vocational indecision (using the total score, the three categories and the ten domains). For each significant relationship, we conducted regression analyses to evaluate the contribution of mixed emotional responses to vocational indecision.

## Results

### TIP-Induced Emotions

**Figure 2.**
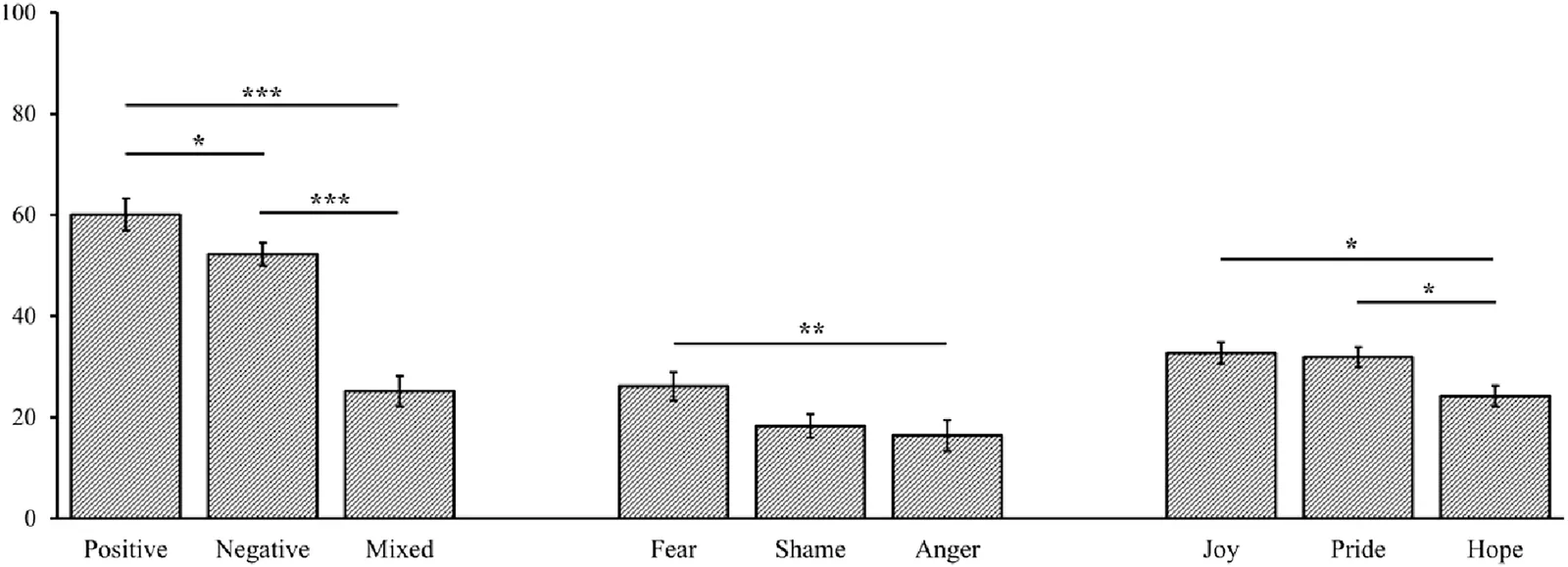
Percentage of TIP Photos Eliciting Emotions. *Note.* Proportion of Positive, Negative and Mixed Emotions Were Calculated Based on the Total Number of Photos Presented (n = 30). Proportions of Individual Emotion Categories (i.e., Fear, Shame, Anger, Joy, Pride, and Hope) Were Computed Relative to the Total Number of Reported Negative or Positive Emotions. Error Bars Represent the Standard Error of the Mean (SEM). Asterisks Indicate *p*-Values for *t*-Tests Comparing Either Emotion Valence (Positive, Negative, Mixed), Negative Emotions (Fear, Shame, Anger), or Positive Emotions (Joy, Pride, Hope). ^*^p < .05, ^**^p < .01, ^***^p < .001

During the task participants were shown professional activity photos and asked to indicate whether imagining themselves performing the depicted activity elicited any positive emotions (specifically joy, pride, hope, or other). In a second round, they were asked whether imagining performing the activity elicited negative emotions (specifically fear, anger, shame, or other). Results show that a high proportion of TIP photos triggered emotional reactions. On average, 87.1% of the 30 photos triggered at least one emotion among the participants; 60.1% triggered at least one positive emotion (either one of the predefined categories or another spontaneously named by the participant), and 52.2% triggered at least one negative emotion. This means that, on average, only 4 out of 30 photos did not elicit any emotional response. Moreover, 25.1% of the photos elicited both a positive and a negative emotion simultaneously for the same person (i.e., mixed emotions). All three emotions occurrences (positive, negative, mixed) were significantly above 0: for positive emotions, *t* (56) = 26.28, *p* < .001, Cohen’s d = 3.48; for negative emotions, *t* (56) = 16.52, *p* < .001, d = 2.19; for mixed emotions, (*t* (56) = 8.46, *p* < .001, d = 1.12). Unsurprisingly, the overall report of any emotion was also significantly above 0 (*t* (56) = 44.66, *p* < .001, d = 5.92). These findings support Hypotheses 1a and 1b. Details of the emotional responses are provided in Figure 2.

Overall, the TIP photos elicited more positive emotions than negative emotions (*t* (56) = 2.17, *p* = .034, d = 0.29). Mixed emotions were significantly less frequent than both positive (*t* (56) = 14.35, *p* < .001, d = 1.90) and negative emotions (*t* (56) = 12.66, *p* < .001, d = 1.68). Regarding the specific emotions reported, 542 occurrences of TIP-induced negative emotions corresponded to the predefined labels: fear (246 times), anger (162 times), and shame (134 times). In addition, participants spontaneously reported 351 other negative emotions, most frequently boredom (129 times) and disinterest (75 times). On the positive emotions side, 921 emotional responses matched the predefined categories: joy (340 times), pride (343 times), and hope (238 times). Another 106 positive emotions were reported outside the predefined categories, the most common being usefulness (33 times), satisfaction (26 times), and amusement (23 times). Full frequency details are available in Table 1.

**Table 1.** Frequency of Predefined Positive and Negative Emotions, and Possible Pairs of Mixed Emotions Elicited by TIP Stimuli, Shown Overall and by RIASEC Domains

	All Domains			R			I			A			S			E			C		
	N	♂	♀	N	♂	♀	N	♂	♀	N	♂	♀	N	♂	♀	N	♂	♀	N	♂	♀
Positive emotions	921	531	375	126	81	44	142	86	54	178	88	85	232	139	89	128	75	51	115	62	52
Joy	340	191	143	33	22	11	32	24	8	90	45	42	68	35	31	64	36	28	53	29	23
Pride	343	202	139	76	48	28	65	38	27	68	35	32	66	45	21	36	20	15	32	16	16
Hope	238	138	93	17	11	5	45	24	19	20	8	11	98	59	37	28	19	8	30	17	13
Negative emotions	542	278	241	123	63	56	77	35	37	89	52	36	98	44	49	72	42	26	83	42	37
Fear	246	111	128	54	21	33	45	19	23	29	13	16	66	29	34	30	17	12	22	12	10
Anger	162	87	62	31	19	9	19	11	6	26	15	10	27	13	12	23	13	7	36	16	18
Shame	134	80	51	38	23	14	13	5	8	34	24	10	5	2	3	19	12	7	25	14	9
Mixed emotions																					
Fear-joy	52	31	20	6	4	2	7	6	1	12	5	7	13	6	6	10	7	3	4	3	1
Fear-pride	63	31	31	18	8	10	14	6	8	7	4	3	12	6	6	6	4	1	6	3	3
Fear-hope	56	25	27	5	2	3	13	5	6	3	1	2	27	13	12	5	3	2	3	1	2
Anger-joy	29	18	10	1	1	0	1	1	0	8	5	3	9	3	5	7	6	1	3	2	1
Anger-pride	29	19	10	6	5	1	5	3	2	7	4	3	4	3	1	3	1	2	4	3	1
Anger-hope	20	14	4	1	1	0	1	1	0	2	0	1	8	6	2	3	2	0	5	4	1
Shame-joy	12	9	3	2	2	0	0	0	0	3	2	1	0	0	0	2	1	1	5	4	1
Shame-pride	14	11	3	5	4	1	1	0	1	4	4	0	1	1	0	2	2	0	1	0	1
Shame-hope	6	2	4	1	1	0	0	0	0	1	0	1	2	1	1	0	0	0	2	0	2

*Note.* Total N reflects the number of reported occurrences across all participants, including those who did not identify as female or male; therefore, Total N may not exactly correspond to the sum of the frequencies for female and male participants.

Among the negative emotions identified *a priori*, fear was reported significantly more often than anger (*t* (56) = 2.86, *p* = .006, d = 0.38). The difference between fear and shame did not reach statistical significance (*t* (56) = 1.79, *p* = .08, d = 0.24), nor did the difference between anger and shame (*t* (56) = −0.50, *p* = .62, d = −0.07). On the positive emotions side, joy and pride were both reported slightly more often than hope (*t-*values ranging from 2.27 to 2.39, *p*-values between .020 and .027, with Cohen’s d around 0.30 and 0.32), but the difference between joy and pride was not significant (*t* (56) = 0.29, *p* = .78, d = 0.04). Across the total of 1710 trials (30 photos x 57 participants), mixed emotional responses were reported in 281 cases, representing 16.48% of all trials. These mixed emotions often reflected the same emotional trends observed in the individual responses. The most frequently reported mixed-emotion pair was fear-pride (63 occurrences), followed by fear-hope (56) and fear-joy (52). Combinations involving anger were also relatively less frequent, notably anger-joy and anger-pride (29 times each), as well as anger-hope (20). Mixed emotional responses involving shame were less common, with shame-pride (14 occurrences), shame-joy (12) and shame-hope (6). Full distributions of each emotional pairing are presented in Table 1.

### TIP-Induced Emotions and Vocational Interests

Table 2 presents the tests of the association between the TIP-induced emotions and vocational interests as inferred from participants’ responses to the TIP images. Results indicate a significant positive correlation between general interest levels and the overall expression of positive emotions during the experience (*r* (55) = .27, *p* = .04). When broken down by RIASEC domain, a similar pattern emerged: for each domain, TIP-induced interest in a domain was positively and significantly associated with positive emotions elicited by TIP images representing that domain (*r* (55) ranging from .30 to .54, all *p* < .05). These results support Hypothesis 2a. More specifically, the emotion of joy was positively and significantly associated with interest in the Social domain, hope with the Artistic domain, and pride with the Enterprising domain, as well as with general TIP-related interest. These findings support Hypothesis 2b.

**Table 2.** Pearson Correlation Between TIP-Induced Emotions and TIP-Induced Vocational Interests

TIP-induced emotions	TIP-induced interests						
	General level of interest	R	I	A	S	E	C
Positive emotions							
All	**.27** ^*^	.14	.15	.06	.15	**.28** ^*^	.05
R	.17	**.45** ^***^	.15	.16	−.02	.10	−.02
I	.07	.18	**.47** ^***^	−.13	.00	−.05	−.19
A	.07	−.08	−.13	**.36** ^**^	.02	.11	−.11
S	**.27** ^*^	.16	.14	.04	**.30** ^*^	.12	.05
E	.17	−.09	−.01	−.14	.14	**.50** ^***^	.10
C	**.29** ^*^	−.05	.02	−.09	.15	.23	**.54** ^***^
Joy	.12	−.09	−.02	−.17	**.27** ^*^	.14	.16
Pride	**.31** ^*^	.22	.24	.02	.15	**.30** ^*^	.04
Hope	.03	.13	.04	**.31** ^*^	−.20	.03	−.14
Negative emotions							
All	.04	−.23	−.16	.05	−.10	.24	**.27** ^*^
R	.08	**−.29** ^*^	−.13	−.10	.04	.20	**.44** ^***^
I	.17	−.14	−.12	.13	.08	.21	**.27** ^*^
A	.03	−.12	.05	−.02	−.18	.12	.24
S	−.04	−.25	−.20	.04	−.19	.25	.19
E	.04	−.14	−.21	.20	−.13	.22	.15
C	−.08	−.10	−.13	.05	−.09	.10	−.05
Fear	.19	−.21	−.04	.09	.07	**.40** ^**^	.20
Anger	−.08	−.16	−.20	.13	−.21	.02	.16
Shame	−.05	−.13	−.13	−.12	−.12	.06	.26
Mixed emotions							
All	.05	−.10	−.12	.17	−.13	**.27** ^*^	.03
R	−.04	.04	−.06	.02	−.11	.03	−.03
I	.09	−.05	−.03	.10	.04	.20	−.03
A	.09	−.07	.02	**.29** ^*^	−.16	.16	.04
S	.07	−.15	−.16	.10	−.04	**.31** ^*^	.11
E	.02	−.15	−.10	.18	−.10	.24	−.03
C	−.04	.01	−.15	.02	−.21	.20	.03

*Note.* The number of individual emotions (Joy, Pride, Hope, Fear, Anger, and Shame) was not broken down by domain due to low frequencies per cell. Bold values indicate significant associations. ^*^p < .05, ^**^p < .01, ^***^p < .001.

Conversely, no significant association was observed between the overall expression of negative emotions and general interest in the TIP photos (*r* (55) = 0.04, *p* = .76). At the RIASEC level, most associations between domain-specific TIP-induced interests and domain-representative negative emotions were non-significant and negative. The only significant exception was the Realistic domain, where a negative association was found (*r* (55) = −0.29, *p* = .03). Thus, Hypothesis 2c is mostly unsupported, apart from this exception. Interestingly, negative emotions elicited by Realistic and Investigative activities were positively associated with interest in the Conventional domain. Among specific negative emotions, only Fear showed a significant positive correlation with interest in the Enterprising domain, lending partial support to Hypothesis 2d. As for mixed emotions, no significant association was found between their overall expression and general interest levels. However, at the RIASEC level, mixed emotions were positively associated with interest in the Enterprising domain. Additionally, mixed emotions in response to Artistic activities (typically joy-fear, cf. Table 1) were significantly associated with interest in the Artistic domain. Surprisingly, mixed emotions toward Social activities (typically hope-fear) were positively associated with interest in the Enterprising domain. These results offer partial support for Hypothesis 2e.

### Dispositional Emotions, TIP-Induced Emotions, and Vocational Indecision

As a preliminary step, we examined descriptive statistics for dispositional emotions and vocational indecision scales (see Table 3). We then tested whether our sample replicated prior findings on the association between dispositional negative affects (PANAS-) and vocational indecision. As expected, PANAS- scores were positively associated with vocational indecision, both at the level of the total score (*r* (51) = .54, *p* < .001), as well as with each of its three main categories: Lack of Readiness (*r* (51) = .51, *p* < .001), Lack of Information (*r* (51) = .41, *p* = .003), and Inconsistent Information (*r* (51) = .38, *p =* .005). A multiple regression using these three indecision categories to predict PANAS- scores confirmed a significant overall model (*F* (3,48) = 7.45, *p* < .001, R^2^ = .32), but only Lack of Readiness emerged as a significant individual predictor (*F* (1,48) = 8.47, *p* = .005, β = .39). Further analyses of this domain revealed that two subscales, Lack of Motivation and Indecisiveness, were significantly associated with PANAS- (respectively *F* (1,48) = 11.11, *p* = .002, β = .36; and *F* (1,48) = 25.63, *p <* .001, β = .56).

**Table 3.** Descriptive Statistics for the Dispositional Affect Scales and Vocational Indecision Scale

	α	N	M	SD	K	S
Dispositional affects						
PANAS-	.76	56	2.21	0.74	0.22	0.80
PANAS+	.72	56	3.37	0.64	0.11	−0.37
Vocational indecision						
CDDQ total score	.88	53	3.62	1.07	−0.93	0.01
Lack of readiness	.56	53	3.85	1.02	0.41	0.62
Lack of motivation	.45	53	2.92	1.48	0.96	1.04
Indecisiveness	.67	53	5.00	1.88	−0.76	−0.40
Dysfunctional beliefs	.54	53	3.63	1.52	−0.46	0.50
Lack of information	.89	53	3.47	1.53	−0.5	0.39
About the process	.79	53	3.71	1.81	−0.83	0.38
About the self	.82	53	3.36	1.92	−0.51	0.69
About the options	.69	53	3.72	1.88	−0.27	0.40
About the sources	.56	52	3.12	1.80	−0.66	0.60
Inconsistent information	.69	52	3.19	1.2	−0.70	0.25
Unreliable information	.70	52	3.90	1.95	−0.93	0.37
Internal conflict	.39	52	3.49	1.32	−0.59	−0.07
External conflict	.85	52	2.18	1.77	1.73	1.67

Regarding dispositional positive affects (PANAS+), results revealed negative associations with overall vocational indecision (*r* (51) = −.41, *p* = .003) and each of the three main categories: Lack of Readiness (*r* (51) = −.37, *p =* .008), Lack of Information (*r* (51) = −.36, *p* = .008), and Inconsistent Information (*r* (51) = −.36, *p =* .01). A regression using these three categories to predict PANAS+ was significant (*F* (3,48) = 4.09, *p* = .012, R^2^ = .20), but no individual predictor uniquely accounted for this effect (*F* (1, 48) = 0.82 to 2.47, *p* = .12 to .37). These findings support Hypothesis 3a, particularly regarding the strong positive relationship between dispositional negative affects and vocational indecision. In testing Hypothesis 3b, PANAS- scores were significantly associated with the number of TIP-induced negative emotions (*r* (54) = .30, *p* = .02). However, PANAS + scores were not significantly related to TIP-induced positive emotions (*r* (54) = .11, *p* = .42), suggesting that situational positive emotional responses to the TIP images cannot be reduced to dispositional positive affects. Thus, Hypothesis 3c is partially supported.

Finally, with respect to Hypothesis 3c, no significant association was found between TIP-induced mixed emotions and overall vocational indecision (*r* (51) = .15, *p* = .29), or with the three categories (*r* ranging from .12 to .21, *p* from .13 to .41), or the ten subscales *(r* ranging from −.02 to .26, *p* from .06 to .91). Similarly, mixed emotions expressed within specific RIASEC domains showed no significant relation to overall vocational indecision (*r* ranging from −.13 to .22, *p* from .12 to .41). An exception was observed for mixed emotions toward the Social domain, which were significantly associated with Inconsistent Information (*r* (50) = .30, *p* = .031). However, regression analyses on this category yielded a non-significant model (*F* (3,48) = 2.33, *p* = .09, R2 = .13), despite a notable correlation between mixed emotions in the Social domain and the internal conflict subscale (*r* (50) = .33, *p* = .002). These findings indicate partial and limited support for Hypothesis 3c.

## Discussion

This study aimed to explore the role of emotions in career choice using an experimental design. Our analyses suggest that imagining oneself engaged in various occupational activities elicits positive, negative, and mixed emotional responses. These emotions are consistently, yet differentially, related to vocational interests. Additionally, our findings replicate previous results linking dispositional affects and vocational indecision, and further extend this line of research by showing a direct relationship between dispositional affects and state-like negative emotional responses.

### Conceptual Implications

The first contribution of this study is to provide a more nuanced understanding of the role of emotions in the career decision-making process. Overall, our results show that expressing positive emotions in response to the TIP photos was associated with higher levels of vocational interest. Moreover, there was a significant positive relationship between expressing interest in a RIASEC domain and experiencing positive emotions toward it. These findings align with those of Puffer (2015), who observed similar results using occupational descriptors. Interestingly, different types of positive emotions were associated with interests in different RIASEC domains. Specifically, expressing joy was linked to Social interests, pride to Enterprising interests, and hope was associated with Artistic interests. These distinctions highlight the specific roles that various positive emotions may play in shaping vocational preferences. They suggest it is not just emotional valence (positive vs. negative) that aligns with vocational motives, but the type of emotions itself. For example, fostering pride may strengthen identification with leadership or competitive roles, while cultivating joy may enhance attraction to prosocial or relational careers, and promoting hope may amplify the desirability of artistic careers. Joy has been defined as “a delight that arises in response to a source of meaning or value in life” (Krumrei-Mancuso, 2020, p. 60). The link between joy and meaning is particularly salient in interpersonal contexts (Chauhan et al., 2020), and joy has been closely associated with altruistic behavior (Zeng et al., 2017).

In contrast, Hope can be defined as “the expectation that one will have positive experiences or that a potentially threatening or negative situation will not materialize or will ultimately result in a favorable state of affairs” (American Psychological Association, 2015, p. 503). In the vocational context, the expression of hope has been suggested as a strategy to cope with the insecurity inherent in artistic careers (Lindström, 2018). In fact, Hoff et al. (2024) highlight a large mismatch between individuals’ interest in artistic careers and the demand from the labor market. Conversely, Thompson and Dahling (2012) show that learning experiences, self-efficacy, and positive professional outcome expectations in the Enterprising domain are more strongly related to perceived ability to obtain power and prestige than in other RIASEC domains. These results could then help explain the observed link between the emotion of Pride—which can be defined as “a self-conscious emotion that occurs when a goal has been attained and one’s achievement has been recognized and approved by others” (American Psychological Association, 2015, p. 829)—and the interest in the Enterprising domain.

A second conceptual implication of this study concerns the importance of context in evaluating emotional reactions to vocational activities, as illustrated by the observed relationships between TIP-induced negative emotions and expressed interest. Emotions are inherently context-dependent, and situational interest has been defined as a “context-specific state of emotional experience” (Su et al., 2009, p. 860). In this study, a negative overall emotional experience with the TIP was positively associated with interest in the Conventional domain. This finding may be explained by the fact that a substantial proportion of participants (31.58%) were already enrolled in apprenticeships in Conventional fields (i.e., as IT specialists, office clerks). The rejection of alternative vocational options may thus reflect a need for cognitive closure, understood as “a desire for definitive knowledge on some issue, for a firm answer to a question and an aversion to ambiguity” (Shiloh et al., 2001, p. 701). Further supporting this idea, we found a positive association between negative emotions toward Realistic activities and interest in the Conventional domain. This may be due to the contrasts in environments: Realistic activities depicted in the TIP photos were mostly outdoor-oriented, whereas Conventional activities were represented in indoor settings, which may align more closely with participants’ preferences or current training experiences. Similarly, negative emotional reactions to Investigative activities—often perceived as more academic—were also associated with higher interest in Conventional fields, possibly reflecting a misalignment between Investigative options and participants’ vocational training context, which favored applied apprenticeship over academic pursuits.

A third conceptual implication is to highlight the role of mixed emotions, and how they may be related to vocational indecision, especially internal conflicts. Parmentier et al. (2021, 2022, 2023) showed that individuals often report conflicting emotions when anticipating career transition. Gati et al. (2010) highlighted that internal conflicts are among the most severe career difficulties, capable of both impeding the decision process and increasing the likelihood of poor career choice. Despite this, career decision-related ambivalence remains understudied, with only a limited number of publications addressing it over the past two decades (see Rochat et al., 2025, for a review). Alarmingly, internal conflicts no longer appear in recent taxonomies of career decision-making difficulties (e.g., S. D. Brown & Rector, 2008; Xu & Bhang, 2019), suggesting a declining emphasis on this critical issue within vocational psychology.

This study thus reinstates the importance of the difficulty of internal conflict in the career decision-making process and emphasizes its emotional nature. In fact, career practitioners surveyed by Gati et al. (2010) hypothesized that internal conflicts were primarily emotional, rather than cognitive in nature. However, to date, the relationship between career decision ambivalence and the presence of conflicting emotions has not been empirically examined. In our study, while mixed emotions were not broadly linked to overall vocational indecision, they were significantly associated with internal conflict within the Social domain. This finding is particularly relevant, as Social interests are projected to be among the most valued in the future labor market—ranking third after Realistic and Enterprising domains (Hoff et al., 2024)—and are disproportionately endorsed by women (Su et al., 2009) and individuals from minority backgrounds (Jones et al., 2021). As such, greater attention to emotional ambivalence in this domain may contribute to more equitable and socially responsive approaches in vocational psychology. In particular, understanding how structural or cultural expectations contribute to emotional ambivalence in these populations could inform more inclusive and nuanced career counseling strategies.

### Methodological Implications

A key methodological contribution of this study lies in the development of an innovative, context-sensitive research protocol that more effectively captures the emotional dimension of career decision-making than traditional approaches. Rather than relying solely on abstract or trait-based self-report measures, we presented participants with photos depicting individuals engaged in a variety of vocational activities and asked them to report their emotional responses in real time. This ecologically valid approach allowed us to examine discrete emotional reactions—both positive and negative—as they occurred in direct relation to specific career-relevant stimuli. In doing so, the study offers a more dynamic, situated understanding of how emotions inform vocational interests and indecision. This approach marks an important step forward in emotion-focused vocational research, providing a framework that better reflects the real-life complexity of career decision-making processes.

### Practical Implications

The major practical implication of this study is the demonstration that emotions are a crucial component in the assessment of career options. These findings are consistent with Phan et al.’s (2019) proposal to integrate emotional symbols, such as emojis, into career assessment to allow for a more nuanced understanding of career clients’ interests. In our experimental setting, participants were not only able to express their level of interest in various vocational activities, but also to identify the emotions these activities elicited. This dual reporting provided richer information about their preferred RIASEC domains (i.e., Social, Artistic, Enterprising, and Conventional). Notably, our results help to reassert the value of negative emotions in the vocational choice process. Rather than viewing them solely as barriers, Meijers (2002) argues that negative emotional experiences can serve as powerful learning opportunities.

The prominence of fear among the negative emotions reported deserves particular attention. In the present context, the fear mechanism pertains to an anticipatory emotion reflecting uncertainty, potential failure, or social evaluation during career-related reflection. This interpretation aligns with the high emotional stakes of vocational choice situations, where success or rejection can strongly affect one’s self-image and perceived future prospects (Ruvolo & Markus, 1992). Interestingly, fear often appeared in mixed-emotion pairs such as fear–pride, fear–hope, or fear–joy, suggesting an ambivalent pattern combining apprehension with motivation or optimism. Future research could explore these emotional blends in greater depth, as they may reflect adaptive mechanisms that sustain engagement in the face of vocational uncertainty. According to this perspective, when clients express negative emotions toward a particular option, career counselors should take these reactions seriously and support clients in exploring their underlying meanings. In line with this, our findings suggest that contextualizing emotional responses—by linking them to specific activities or imagined scenarios—can enhance career self-awareness and support more informed decision-making.

As a result, career counselors might consider routinely asking clients not only to rate their interest in different careers or tasks, but also to identify the emotions these options evoke. Providing a structured list of emotions may assist clients who struggle to label their feelings, thereby improving their emotional intelligence (or competency)—a capacity that has been linked to several positive vocational outcomes, such as decisiveness and career decision-making self-efficacy (see Pirsoul et al., 2023, for a meta-analysis). This kind of emotion-focused exploration may also reveal the presence of ambivalence or mixed feelings, which, as our study suggests, are significantly associated with vocational indecision. Engaging with such ambivalent reactions in a constructive manner may help clients gain insight into internal conflicts that may otherwise remain unspoken or unexplored. While mixed emotions are often seen as obstacles to clarity (e.g., Kasperzack et al., 2014), recent work by Rochat et al. (2025) highlights their potential to foster creative and adaptive career choices. In fact, in today’s volatile and complex labor market, Ashford et al. (2018) argue that the ability to tolerate and navigate ambivalence is essential to career adaptability and long-term success.

### Limitations and Future Direction

Several limitations of this study should be acknowledged. First, although our experimental design allowed for the elicitation of multiple discrete emotions, the emotion list was limited to a predefined set of six (three positive and three negative emotions). While additional emotions were occasionally mentioned by participants, they were not assessed in a systematic way, limiting the granularity and depth of our emotional analysis. Future studies could benefit from employing a broader and more nuanced set of emotions, including affects potentially relevant to career decision-making—such as boredom, disgust, curiosity, or anxiety. For instance, Young et al. (1997) identified “boring” and Puffer (2015) “blandness” as recurrent emotional responses leading individuals to reject certain vocational options. Such emotions, which we also observed informally in our study, could be explored in greater detail to clarify their relationships with vocational interest, RIASEC domains, and indecision. Similarly, as “fear” appears to be the most prevalent negative emotions identified in the present study regarding career options, it would be interesting to explore this emotion in more detail.

Second, the context-specific nature of our experimental procedure, while ecologically valid, also limits the generalizability of our findings. Emotions were elicited in a structured setting using standardized photos, which may not fully reflect the complexity and richness of real-life vocational experiences. Broader structural and socio-cultural facets of working life can also significantly shape young people’s emotional experiences related to occupations, such as the perceived relational atmosphere, organizational norms, risks, fairness in working hours, or levels of autonomy and social recognition (Masdonati, Fedrigo, & Zufferey, 2022). These factors, which go beyond the activities themselves, may complicate the interpretation of emotions by embedding them within wider systems of meaning and power (Masdonati, Fournier, & Lahrizi, 2022), and contribute to explaining why the female participants expressed fewer emotional reactions (both positive and negative) than male participants across the experiment. Beyond individual emotional responses to occupational activities, it is important to recognize that such reactions are embedded within broader structural and social contexts., Indeed, the individuals depicted in the images were predominantly of North-Caucasian ethnicity, potentially introducing implicit bias or limiting identification, particularly among those from more diverse backgrounds. 

Moreover, the emotional meaning of work is shaped by how professions are valued within society, by gendered and socioeconomic expectations, and by the perceived attainability of different career paths (Gottfredson, 2002). For young people facing educational or vocational difficulties, emotions toward specific occupations may thus reflect internalized social norms and constrained opportunities rather than pure personal preference. This broader perspective highlights that interpreting emotional responses to work-related stimuli requires considering both psychological and structural determinants of meaning. Future studies should consider using a demographically diverse stimulus set to enhance ecological and cultural validity. Third, we did not assess participants’ emotional regulation abilities or emotional intelligence, both of which influence how participants experience, label, interpret and respond to career-related emotions (Pirsoul et al., 2023). These individual differences could contribute significantly to variability in emotional reactivity and the decision-making process. Including such measures in the future would allow for a more nuanced understanding of the emotional mechanisms underlying vocational development. Similarly, future studies could focus on the links between emotional responses and career indecisiveness which denotes “more pervasive, severe, and chronic difficulties in making career decisions” than vocational indecision (Saka & Gati, 2007, p. 341).

Fourth, the sample lacked heterogeneity in youth trajectories, as the majority of participants were enrolled in integration programs. While this population is highly relevant for research on vocational indecision, it represents a specific segment of the youth population—one often marked by institutional support, educational discontinuities, or constrained opportunities. Although these situations are of concern in many European countries (see Hofmann et al., 2021), these characteristics may not be representative of youth in academic environments or those following more autonomous career paths, limiting the applicability of our findings to broader populations. Future studies should seek to include more diverse educational and developmental profiles to examine whether the observed emotional patterns and decision-making dynamics generalize across different youth subgroups. Fifth, Podsakoff et al. (2003) suggest counterbalancing the order of questions to control for priming effects in behavioral research. Accordingly, future research could consider reversing the order of the second and third parts of the experimental the protocol to assess potential priming or fatigue effects and strengthen the internal validity of the design. Finally, all emotional responses were self-reported, which, while common in psychological research, may introduce biases or limitations in emotional accuracy (Pirsoul et al., 2019). To enhance the validity of emotional assessment, future research could integrate physiological markers—such as heart rate variability or skin conductance—to better capture emotional intensity and regulation beyond verbal reports (Rochat & Banet, 2020). A multimodal approach would allow researchers to explore implicit affective processes that may unconsciously influence vocational preferences and choices.

## Conclusion

This study aimed to examine the role of emotions in vocational interest and indecision by exploring emotional reactions to professional activities depicted in photos. Our findings confirm that such activities elicit a range of emotional responses—positive, negative, and mixed—and that these emotions are meaningfully related to vocational interests. Notably, both distinct positive and negative emotions were independently associated with interest in particular RIASEC domains—joy with Social interests, hope with Artistic interests, pride with Enterprising interests, and fear or anger with increased interest in Realistic and Conventional domains. Dispositional affects were also associated with vocational indecision, particularly in relation to readiness-related difficulties. Emotional ambivalence may play a role in specific vocational contexts, particularly where values or social expectations create a tension. These findings suggest that emotionally complex or ambivalent reactions can carry important information for vocational exploration. Future studies may build on these findings by examining how emotional competencies and contextual diversity shape career decision-making in youth.

## Data Availability

The data that support the findings of this study are available from the corresponding author, SR, upon reasonable requests.*
